# The Low-Carbohydrate Diet: Short-Term Metabolic Efficacy Versus Longer-Term Limitations

**DOI:** 10.3390/nu13041187

**Published:** 2021-04-03

**Authors:** Thomas M. Barber, Petra Hanson, Stefan Kabisch, Andreas F. H. Pfeiffer, Martin O. Weickert

**Affiliations:** 1 Warwickshire Institute for the Study of Diabetes, Endocrinology and Metabolism, University Hospitals Coventry and Warwickshire, Clifford Bridge Road, Coventry CV2 2DX, UK; T.Barber@warwick.ac.uk (T.M.B.); drpetrahanson@gmail.com (P.H.); 2 Division of Biomedical Sciences, Warwick Medical School, University of Warwick, Coventry CV2 2DX, UK; 3 NIHR CRF Human Metabolism Research Unit, University Hospitals Coventry and Warwickshire, Clifford Bridge Road, Coventry CV2 2DX, UK; 4 Department of Endocrinology, Diabetes and Nutrition, Campus Benjamin Franklin, Charité University Medicine, Hindenburgdamm 30, 12203 Berlin, Germany; stefan.kabisch@charite.de (S.K.); afhp@charite.de (A.F.H.P.); 5 Deutsches Zentrum für Diabetesforschung e.V., Geschäftsstelle am Helmholtz-Zentrum München, Ingolstädter Landstraße, 85764 Neuherberg, Germany; 6 Centre for Sport, Exercise and Life Sciences, Faculty of Health & Life Sciences, Coventry University, Coventry CV2 2DX, UK

**Keywords:** Low-Carbohydrate Diet, Type 2 Diabetes Mellitus, fat mass, ketogenesis

## Abstract

Background: Diets have been a central component of lifestyle modification for decades. The Low-Carbohydrate Diet (LCD), originally conceived as a treatment strategy for intractable epilepsy (due to its association with ketogenesis), became popular in the 1970s and since then has risen to prominence as a weight loss strategy. Objective: To explore the efficacy, limitations and potential safety concerns of the LCD. Data Sources: We performed a narrative review, based on relevant articles written in English from a Pubmed search, using the terms ‘low carbohydrate diet and metabolic health’. Results: Evidence supports the efficacy of the LCD in the short-term (up to 6-months) for reduction in fat mass and remission of Type 2 Diabetes Mellitus (T2D). However, the longer-term efficacy of the LCD is disappointing, with diminishment of weight loss potential and metabolic benefits of the LCD beyond 6-months of its adoption. Furthermore, practical limitations of the LCD include the associated restriction of food choices that restrict the acceptability of the LCD for the individual, particularly over the longer term. There are also safety concerns of the LCD that stem from nutritional imbalances (with a relative excess of dietary fat and protein intake with associated dyslipidaemia and increased risk of insulin resistance and T2D development) and ketotic effects. Finally, the LCD often results in a reduction in dietary fibre intake, with potentially serious adverse consequences for overall health and the gut microbiota. Conclusions: Although widely adopted, the LCD usually has short-lived metabolic benefits, with limited efficacy and practicality over the longer term. Dietary modification needs tailoring to the individual, with careful a priori assessments of food preferences to ensure acceptability and adherence over the longer term, with avoidance of dietary imbalances and optimization of dietary fibre intake (primarily from plant-based fruit and vegetables), and with a posteriori assessments of the highly individual responses to the LCD. Finally, we need to change our view of diets from simply an excipient for weight loss to an essential component of a healthy lifestyle.

## 1. Introduction 

The global obesity epidemic poses a major threat to our health, with much associated chronic ill health that includes >50 separate obesity-related co-morbidities [1,2]. Cardio-metabolic dysfunction underlies many obesity-related conditions, including Type 2 Diabetes Mellitus (T2D), other features of the metabolic syndrome such as dyslipidaemia and hypertension and obesity-related malignancies that include endometrial carcinoma [3]. Global obesity also contributes to substantial socio-economic costs [4]. There is an urgent need to address global obesity through efficacious and long-term strategies and preventive measures.

Traditionally, there is division of obesity management into three distinct phases: lifestyle, pharmacotherapy and bariatric surgery. Currently, bariatric surgery provides our best evidence for long-term effective weight loss in obesity [5]. Although pharmacotherapies for obesity have a chequered history, there is much promise for the future based on data from some of the newer classes of therapies for T2D, including incretin-based hormonal suppressants of hunger [6,7]. Regarding lifestyle strategies for obesity, although usually considered as an early strategy for weight loss, healthy lifestyle behaviours should form a foundation for weight loss, applicable throughout its management and complementary to other strategies.

Lifestyle is an umbrella term that incorporates all aspects of healthy behaviours including sleep quality and sufficiency, physical activity, smoking cessation and the optimization of mental and emotional health. Attention to diet forms a major component of a healthy lifestyle. There are a myriad of diets available, based on multiple variables. These include the combinations of macronutrients, the duration and timing of meals, the duration and periodicity of fasting periods, chewing time, the speed of eating, the duration of the diet, the types of cooking techniques and even the emulation of previous eras such as the ‘Paleolithic Diet’. Amongst all available diets though, the Low-Carbohydrate Diet (LCD) features prominently. The LCD, conceived a century ago as a means of inducing ketonaemia as a treatment strategy for intractable epilepsy [8], has only been re-purposed for weight loss and metabolic benefits for the second half of its history, in response to the burgeoning global obesity problem. Indeed, even prior to the discovery of insulin 100-years ago, and before the conception of the LCD as a treatment for intractable epilepsy, severe restriction of carbohydrate intake was advocated for the treatment of Type 1 Diabetes Mellitus (T1D) [9]. In this concise review, we consider the definition, rationale and patient selection for the LCD. We also critically review current literature on the metabolic efficacy of the LCD, and consider its limitations and potential safety concerns. Finally, we outline the role of the LCD in the context of a healthy lifestyle program and the future of the LCD. 

## 2. Methodology

We performed a narrative review of the current literature. We used Pubmed for this purpose. The search terms included ‘Low carbohydrate diet’ in combination with the following: ‘metabolic health, definition, physical activity, limitations, nutrition, ketosis, hyperuricaemia, inflammation and microbiota’. We chose published studies for inclusion in our narrative review based on their relative size, novelty and perceived clinical relevance as assessed by the authors; and related review articles, including systematic reviews and meta-analyses. We only considered articles written in English, with no restrictions on the date of publication.

## 3. Low-Carbohydrate Diet: Definition, Rationale and Patient Selection

The definition of the LCD varies between studies, and includes a diet that contains <130 g of carbohydrate per day (or <26% carbohydrate from a 2000 Kcal/day diet) [10]. However, perhaps the most widely used definition of the LCD is based on the percentage of total daily calories (<20%) derived from carbohydrates, with a relatively high proportion of daily caloric intake from fats (55–65%) and protein (25–30%) [11]. The dietary composition of the LCD differs markedly from the Low-Fat Diet (LFD) in which only 20–30% of total daily calories stem from dietary fats, with 55–65% of daily calories derived from carbohydrates and 15–20% derived from protein [11]. These definitions of the LCD and LFD highlight a key underlying problem inherent to any study on dietary modification: the impossibility of exploring the effects of macronutrient changes in isolation. Our diets contain a complex array of macronutrients. If there is a reduced intake of one macronutrient (such as carbohydrates), then there must be a relative increase in the daily caloric contribution from other macronutrients (such as fats and protein), to compensate for the reduction in carbohydrate intake. Therefore, it is important to emphasize that many dietary interventions, including the LCD, are actually more complicated than their titles otherwise suggest. This awareness is important when assessing the outcomes of diets such as the LCD. 

Restriction of dietary carbohydrate intake has a long history, including as a treatment strategy for T1D prior to the discovery of insulin [9]. Indeed, in 1863, Edward Banting commented on his own personal experience of using dietary carbohydrate restriction for weight loss (from 202 to 156 pounds): “*the great charms of the system are that you are never hungry and its effects are palpable within a week of trial and creates a natural stimulus to persevere a few weeks more*”. The original LCD (with relatively high dietary intakes of fat and protein) was conceived 100 years ago in 1921 as a means of inducing ketogenesis, as a non-pharmacological treatment option for intractable childhood epilepsy [8,12]. In recent years, variations of this low-carb ‘ketogenic’ diet (defined as ketosis in the context of the LCD) have improved its palatability and tolerability, and thereby widened its availability to a larger group of patients, including adults who have failed to respond to multiple anticonvulsant therapies and who are not surgical candidates [12]. Interestingly, despite its use as a therapeutic option for intractable epilepsy for a century, the underlying mechanism(s) whereby the low-carb ketogenic diet improves health outcomes in people with epilepsy remains unclear [12]. Hypotheses include effects on mitochondrial function, changes in neurotransmitter release and neuronal function that stem from the effects of ketone bodies (including enhanced membrane potential hyperpolarization, increased γ-aminobutyric acid [GABA] synthesis and reduced release of glutamate), and the anticonvulsant effects of glucose stabilization and fatty acids [12]. The low-carb ‘ketogenic’ diet may also inhibit the mammalian target of rapamycin (mTOR) [12,13,14]. For the first half of its history, the use of the low-carb ‘ketogenic’ diet was mainly for the purpose of managing intractable epilepsy. With the burgeoning global problem of obesity in the 1970s, there was a re-purposing of the LCD for a very different indication: that of weight loss. The concept of the LCD (associated with relatively high intake of protein and fats) for the purpose of weight loss and weight maintenance was popularized in the 1970s by Dr. Robert C Atkins [15]. Since then, and over the second half of its history, the LCD as a lifestyle strategy for weight loss has become prominent, and now far out-weighs its original purpose as a treatment option for intractable epilepsy. 

The rationale of the LCD as a weight loss strategy stems from the Carbohydrate-Insulin Model (CIM) of obesity. This suggests that high-carbohydrate diets (including refined sugar and starches) stimulate post-prandial hyperinsulinaemia that in turn promotes the deposition of calories within fat cells, rather than the alternate fate of oxidation within lean tissues, with consequent weight gain (including effects on increased hunger and suppressed metabolic rate) [16]. Of all the many influences on insulin secretion, dietary carbohydrate has the most potent effect, and varies according to the amount and type of carbohydrate [16]. Although protein also stimulates insulin secretion, there is tempering of the effects of protein-induced hyperinsulinaemia by the concomitant secretion of glucagon. Conversely, dietary fat has little direct effect on the release of insulin [16]. Following adoption of the LCD, two key metabolic processes ensue: gluconeogenesis and ketogenesis [17]. A diminished supply of glucose to the muscles, brain and liver results in a reduction in the synthesis and storage of glycogen, and therefore a reduced capacity for glycolysis. Due to the reciprocal and inverse relationship between glycolysis and gluconeogenesis, diminishment of the former results in augmentation of the latter [17]. The process of gluconeogenesis utilizes certain amino acids (alanine and glutamine), glycerol and lactic acid as substrates [18]. Given the limited supply of these substrates, continuous gluconeogenesis over a prolonged period of many hours may be insufficient to provide the body’s needs for glucose. In this scenario, ketogenesis occurs with the synthesis of ketone bodies as an alternate fuel source to glucose [17]. Low levels of serum insulin that associate with the LCD result in lipolysis with an elevated supply of fatty acids, with conversion to acetoacetic acid and the ketones β-hydroxybutyric acid and acetone [17]. Based on these physiological insights, adoption of the LCD would reduce levels of plasma insulin, and promote the oxidation of ingested calories within lean tissues, with reduced storage as fat and favourable effects on appetite and metabolic rate.

To appreciate further the metabolic benefits of the LCD, it is instructive to consider the metabolic sequelae of adopting a high carbohydrate diet (HCD) that results in increased insulin secretion, that in turn partitions energy away from oxidation and towards storage depots such as fat deposition within adipose tissue [17]. In response to the brain’s perception of ‘cellular internal starvation’, there is enhancement of appetite and suppression of metabolic rate [17]. However, an alternate explanation for weight gain with a HCD simply invokes the pleasant taste of sweetness and its inherent hedonic effects that in turn drives us to eat more. The underlying mechanisms that link the HCD with resultant weight gain may implicate appetite control, the hedonic centres, metabolic rate, insulin-mediated energy partitioning or a combination of all of these factors. Whatever the actual mechanisms, the literature provides clear consensus on the metabolic effects of diets according to carbohydrate content: HCDs promote weight gain and metabolic dysfunction, and LCDs promote weight loss (at least in the short-term) and optimization of metabolic functioning. 

To summarize this section, the LCD is defined as a diet that has a low proportion of daily calories (<20%) derived from carbohydrates. LCDs therefore also contain a relatively high proportion of calories derived from dietary fat and protein, which hampers any attempt to explore the effects of low dietary carbohydrate ingestion in isolation. Although originally conceived as a treatment for intractable epilepsy, the primary application of the LCD currently is to facilitate weight loss and metabolic improvements in people with obesity, including obesity-related co-morbidities such as T2D. In this review, we focus on the metabolic effects of the LCD in these scenarios. Ultimately, there is individualization of the implementation of dietary interventions such as the LCD, according to their rationale and purpose. It is important to consider carefully the available evidence when choosing appropriate dietary interventions such as the LCD, including metabolic efficacy and safety.

## 4. Metabolic Efficacy of the LCD

Adoption of the LCD results in a reduced supply of carbohydrates to the liver that in turn causes a reduction in the conversion of excessive carbohydrates into fatty acids and enhanced lipolysis. There is also a reduction in the levels of plasma insulin that releases the drive to store fat within adipose tissue, that ultimately manifests in progressive loss of body fat [17]. Accordingly, reduced fat mass represents a key metabolic benefit and rationale for the adoption of the LCD. Indeed, at least over the short-term, the LCD appears to result in some superior reduction in body fat mass. The effects of the LCD and Very Low-Carbohydrate Diet (VLCD) on body composition was reported based on a meta-analysis of 14 randomized controlled trials (RCTs) that included >1400 obese individuals, in which there was an overall greater reduction in fat mass of 0.77 Kg when compared to the LFD [19]. In the sub-group with LCD over 12-months, the additional reduction in fat mass was 0.57 Kg [19]. 

Despite these promising data on improved fat mass for the short-term efficacy of the LCD, longer-term data are disappointing. Other meta-analyses based on longer-term randomized weight loss trials that compare LCD with traditional calorie-restricted (low-fat) diets show that although LCDs associate with greater weight loss in the short-term, this advantage of the LCD either disappears or diminishes after 1 year [20,21,22,23]. The reduced efficacy of the LCD on weight loss over the longer-term suggests the emergence of some mitigating compensatory mechanism [24]. One hypothesis is that the reduced glycogen stores that associate with the adoption of the LCD [25] result in reduced physical activity and increased fatigue [24], with consequent reduced energy expenditure. However, no consistent evidence from human-based studies supports any assumption that dietary composition and quality has an appreciable impact on physical activity, performance or endurance in people adopting dietary modification (including the LCD) for the purpose of weight loss [26,27,28]. There is also controversy regarding the effects of the LCD on the capacity for physical activity in sportsmen [29,30,31]. In one fascinating study on *Drosophila melanogaster* that compared a standard diet and LCD for 9-days, although activity was unaffected by the diet, there was a relative reduction in overall metabolic rate in the LCD group. Although caution is required when extrapolating data from *Drosophila* into humans, suppressed overall metabolic rate may represent one possible factor that mitigates the longer-term success of the LCD for effective reduction in body fat [24].

The potential metabolic benefits of the LCD extend well beyond reduced fat mass, including for example potential improvement in the future risk of cardio-vascular disease in patients with pre-diabetes and T2D [32,33]. Through a combination of reduced supply of carbohydrates to the liver and suppressed plasma levels of insulin, the LCD associates with improved (pre-prandial) insulin sensitivity and glycaemic control. Overall, fasting insulin sensitivity improves over the short-term in response to the LCD, but the effect of the LCD on fasting insulin sensitivity diminishes over the longer-term, in tandem with the diminished longer-term effects on body weight loss [32,33]. (Unfortunately, limited data exist for the effects of the LCD on post-prandial insulin sensitivity). In a systematic review and meta-analysis of RCTs on the efficacy of the LCD versus a normal or high-carbohydrate diet in patients with T2D from 9 studies and >700 individuals, there was a significant reduction in HbA1C of 0.44% for the LCD group [34]. The LCD group also had a significant reduction in plasma triglyceride level of 0.33 mmol/L but no significant change in total or LDL cholesterol. Furthermore, although the LCD had an initial reducing effect on body weight, over the longer term (as with outcomes from the general obese cohorts outlined above), there was no significant effect of the LCD on body weight [34]. In a separate study on adoption of the LCD in obese patients with T2D, there was a short-term improvement in insulin sensitivity, optimization of glycaemic control and lipid profile, a spontaneous reduction in energy intake and weight loss (accounted for by the reduced caloric intake) [35]. Furthermore, in a recent meta-analysis of the LCD in patients with T2D on >1350 participants, it was demonstrated that compared with control diets at 6-months, the LCD achieved higher rates of remission from T2D (data on T2D remission at 12-months were sparse), and improvements in weight loss, fasting insulin sensitivity and triglycerides that diminished at 12-months [10]. Finally, the effects of the LCD on non-alcoholic fatty liver disease (NAFLD) remains incompletely understood. Although human data show association of the LCD with a reduction in intra-hepatic triglyceride content, longer-term maintenance of a ketogenic diet in mice actually stimulates the development of NAFLD [36]. 

**Figure 1:**
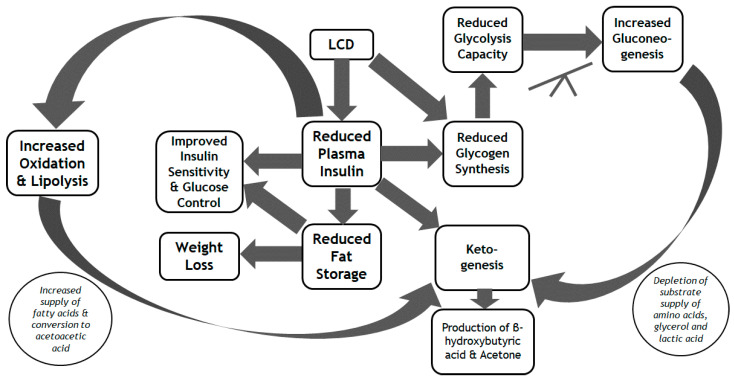
Overview of the short-term metabolic benefits of the Low-Carbohydrate Diet (LCD).

Despite the clear metabolic benefits of the LCD for patients with T2D at least in the short-term, there remains controversy in the literature regarding the relative benefits of the LCD versus other dietary modifications in T2D. In one systematic review and meta-analysis that compared the metabolic effects of the LCD vs. HCD in adults with T2D, the proportion of daily energy provided by carbohydrate intake was not an important factor in the response to dietary management [37]. Furthermore, in a recent meta-analysis, there was an inverse association between T2D incidence and increased intake of whole-grain, and a direct association with the intake of sugar-sweetened beverages [38]. There is also contention regarding the origin of the metabolic benefits of the LCD, with potential benefits from the diet itself, and the indirect metabolic effects that stem from reduced hunger and associated weight loss [39]. It seems likely that a combination of these factors underlies the metabolic benefits of the LCD (at least in the short-term), an overview of which we present in Figure 1.

Although the adoption of the LCD seems a sensible lifestyle approach, and one corroborated by scientific evidence for the short-term promotion of weight loss and improved metabolic status, it is important not to overlook the potential benefits of other types of dietary modification for effective weight loss and metabolic improvements. In one meta-analysis of 32 controlled feeding studies in diet-induced obese mice in which there was isocaloric substitution of carbohydrate for fat, both energy expenditure and loss of fat mass were significantly greater with lower fat diets [40]. Although we should exercise caution when extrapolating rodent data to humans, the LFD in humans also associates with effective weight loss [11]. Furthermore, there are potential practical problems and safety concerns that limit the effective and longer-term adoption of the LCD, explored in the next sub-sections. Therefore, it is important not to choose the LCD by default, but rather consider dietary and lifestyle options from a holistic perspective, tailored to each individual. 

## 5. Limitations of the LCD

Any successful behavioural change requires sustained focus, drive, grit and determination. Although habitualization is possible, behavioural change over the longer term is often challenging. All of us experience life events that demand our focus and attention and may dominate our emotions temporarily. It is at such moments that maintained focus on healthy behaviours including dietary modification becomes particularly challenging, and where many of us falter. The longer-term adherence to diets such as the LCD is no exception. Indeed, poor adherence to dietary recommendations is a major reason for the limited efficacy of dieting per se [41]. The longer-term success of any dietary modification depends on its acceptability to the individual and influenced by its practical applicability and, importantly, the enjoyment associated with its adoption. Unfortunately, much of the pleasurable hedonic effects of eating stem from the ingestion of carbohydrate, and particularly the combination of carbohydrates and fat (that never occurs naturally). Sweet foods are palatable. This poses a problem for the LCD, with potentially reduced palatability of foods that are low in carbohydrates and associated reduced enjoyment from eating contributing to the longer-term problem of adherence to the LCD. Furthermore, the successful adoption of the LCD often results in severe limitation of food choices, with the attendant risk of inadequate nutrition. This further hampers sustained adherence to the LCD [11].

The achievement and maintenance of a healthy body weight is unlikely to occur unless the lifestyle opted for (including dietary modification and physical exercise) are enjoyable [42]. Given individual preferences for food, it follows that the choice of diet should be tailored to the individual [42]. There should be encouragement of changes to the diet that are acceptable to the individual and that can be maintained [42]. Attempts to follow a specific diet that is unacceptable or unpleasant for an individual is unlikely to be successful, and may actually be de-motivating and have a negative impact on self-esteem, self-confidence and likelihood of engagement with other healthy lifestyle behaviours. Therefore, prior to advocating any diet, a carefully elicited dietary history is required, including the types of foods and recipes that the individual finds acceptable and enjoyable. In some cases, a modified LCD may be required, that maintains some elements of the LCD, but may be tempered to improve its acceptability to the individual and therefore to optimize the likelihood of its longer-term success. 

In addition to the acceptability and palatability of the LCD, a further potential limitation relates to its possible hampering of athletic performance. In one study on the effects of the LCD (with associated high-fat diet [HFD]) in elite endurance athletes, in contrast to diets that provide high-carbohydrate availability, training with the LCD impaired athletic performance despite improved peak aerobic capacity [30]. However, as alluded to earlier, there is relatively little evidence from the literature to support any appreciable effects of the LCD on physical endurance and performance for most people who adopt this diet. Furthermore, elite athletes should not represent a standard model from which to judge the physical effects of the LCD in most people who adopt it. Nevertheless, when choosing an appropriate diet, it is important to consider not just its metabolic effects and weight loss potential, but also the potential impact on physical activity, and occasionally and where relevant, athletic performance. Although optimized athletic performance may not be a priority for many of us, the data outlined here nonetheless highlight the importance of a tailored approach to the individual, including consideration for the minority with a focus on athleticism. 

## 6. Potential Safety Concerns of the LCD

The longer-term metabolic and weight-losing efficacy of the LCD, its acceptability to the individual and associated limitations are important considerations. However, an even greater concern relates to the potential safety concerns of the LCD that originate from multiple sources (outlined in Table 1). 

Nutritional Deficiencies: In patients with Type 1 Diabetes Mellitus, adoption of the LCD may result in an increased risk of severe hypoglycaemia, that is resistant to the effects of glucagon due to the associated depletion of glycogen stores [43]. Apart from glycogen deficiency, other nutritional deficiencies associated with the LCD stem from restrictions of dietary choice (and therefore the risk of a nutritionally imbalanced diet), compounded by a concomitant drive to lose weight with additional deliberate restriction of caloric intake. Accordingly, the longer-term adoption of the LCD (usually following at least 3-months) can result in dietary deficiencies of minerals, vitamins and trace elements and associated problems with bone health, renal calculi and occasionally growth failure [12]. A relative paucity of dietary fibre intake in the LCD (from the predominance of animal-based high fat and high protein foods, and relative lack of plant-based foods that contain carbohydrates in the form of dietary fibre) may cause constipation and other health problems [44]. Over the longer term, these imbalances of macronutrient intake can have potentially deleterious effects that stem from relatively high dietary intakes of fat (such as dyslipidaemia [12]) and protein (such as impaired glomerular filtration rate demonstrated in women with mild renal impairment, but not in healthy subjects with eGFR > 60 mL/min/1.73 m^2^ [11,45,46,47]). Furthermore, diets that have both low and high percentages of carbohydrates associate with increased mortality, with minimal mortality risk at 50–55% carbohydrate intake (with a plant-based diet) [48]. A relative lack of plant-based foods (including vegetables and legumes) in the LCD may worsen mortality from a reduced intake of essential micronutrients such as polyunsaturated fatty acids (PUFA) [49,50].

Ketosis: To complement the nutritional problems that associate with longer-term adoption of the LCD, there are also potential safety concerns from the effects of persistent and sustained ketosis. In the short-term, these include potential gastro-intestinal symptoms such as vomiting, diarrhoea, obstipation, gastroesophageal reflux and hypoglycaemia [12]. The longer-term effects of sustained ketosis on the body are incompletely understood [11]. However, ketosis can result in released calcium stores to neutralize the ketones, with a theoretical risk of osteoporosis and nephrolithiasis [11]. Interestingly, epidemiological data reveal that higher protein intake actually associates with reduced bone loss [51]. In extreme and rare cases, carbohydrate restriction may even precipitate ketoacidosis [52].

High-Protein Diet: The LCD associates with intake of a relatively high proportion of dietary protein (to compensate for a relative lack of carbohydrate as a source of energy). There is controversy in the literature regarding the health effects of a high-protein diet. Over the longer term, there are reported unfavourable metabolic effects of a diet that is proportionately high in protein, including association with dysglycaemia [53,54]. In a large prospective cohort study with 1-year follow-up, consumption of energy from protein at the expense of energy from fat or carbohydrates actually increased the risk for the development of T2D [53,54]. Conversely, a recent guidelines publication by the Diabetes and Nutrition Study Group (DNSG) justified higher protein diets (up to 20% and 1.3 g/Kg body weight) for the treatment of T2D, providing normal renal function [47]. Interestingly, there appears to be a contradiction between the epidemiological and interventional studies on high-protein diets, with the latter (20–30% daily energy requirements from dietary protein in the context of a healthy dietary intake of fats, fibres and vegetables) showing consistently favourable metabolic outcomes and biomarkers. Such interventional data include the ProFiMet study with the combination of a high-protein diet with fibre intake [55], and a series of reported studies on the metabolic benefits of high-protein diets on improvements in hepatic fat content, inflammatory profiles and oxidative stress, including in people with T2D and obesity [56,57,58,59]. A likely explanation for the apparent disconnect between the metabolic data from epidemiological and interventional studies on high-protein diets is that the former often fail to correct for lifestyle variables associated with a high dietary protein intake, with a relative lack of plant-based foods and physical inactivity. Conversely, interventional studies on high-protein diets (including those discussed here) usually combine these with a healthy lifestyle program (including a healthy diet), with recent meta-analyses that reveal improvements in most metabolic outcomes [60]. Therefore, based on current evidence, high-protein diets per se do not appear to influence metabolic health, but rather the healthiness of their associated diet and other lifestyle behaviours. 

Hyperuricaemia and Inflammatory Effects: A further theoretical risk of the LCD includes hyperuricaemia (with associated gouty arthritis and uric acid nephrolithiasis) from excessive conversion of purines from animal proteins following adoption of the LCD (with relatively high dietary intake of proteins) [11,61]. However, it is noteworthy that purines also originate from plant-based foods that contain lots of DNA such as cabbage, and that fructose represents an important source of uric acid formation [62]. Finally, theoretically the LCD may promote inflammatory effects through a relative increase in saturated fat intake (possibly mitigated through associated weight loss). In fact, evidence suggests either no appreciable effects [63] or improvements in inflammatory status (at least in the short term) from the LCD [64,65,66,67], and associated improvements in the risk factors for cardiovascular disease [68]. Future longer-term prospective studies should explore the longer-term effects of the LCD on cardio-vascular events. 

Mitigation of some of the nutritional and metabolic problems associated with the LCD could occur through the concomitant optimization of dietary fibre intake. Our own group showed that the ingestion of *insoluble* and non-fermentable cereal fibres (derived from oat or wheat extracts and whole grain products) rather than soluble and highly fermentable types of dietary fibre, associates with reduced risk for the development of T2D and improved insulin sensitivity [55,69,70]. We have proposed a likely mechanistic explanation for the association between dietary fibre intake and improved insulin sensitivity to implicate the reduced absorption of dietary protein and modulation of the amino acid metabolic signature [70,71]. The combination of the LCD with optimized dietary fibre intake may therefore help to reduce the potential detrimental metabolic effects (including insulin resistance) of excessive protein intake, and may reduce the risk of hyperuricaemia. However, this strategy is problematic given that most carbohydrates occur within plant-based foods (in the form of dietary fibre), and the LCD is therefore typically low in dietary fibre. Dietary restriction of fermentable and poorly absorbed carbohydrates (as occurs in the LCD) can have beneficial effects in patients with symptoms of Irritable Bowel Syndrome and small intestinal bacterial overgrowth, in whom reversal of this dietary strategy may therefore worsen such symptoms [72,73]. Furthermore, supplementation of the LCD with fibre-rich foods may potentially exceed the daily limit of carbohydrates allowable for the LCD. To address these problems, supplementation of the LCD with insoluble fibre-rich foods such as cereal fibre (that tends to have a lower carbohydrate content than its counterpart, soluble fibre) provides a potential solution, and would enable the adoption of the LCD whilst also maintaining adequate dietary fibre intake [74,75,76]. Furthermore, fibre-rich vegetables contain relatively low levels of carbohydrate, primarily soluble and metabolically ineffective fibre and essential micronutrients (likely accounting for their health benefits), and as such provide an excellent accompaniment to the LCD. Additional strategies include fibre supplementation and fortification of the LCD. 

As outlined, the main safety concerns regarding adoption of the LCD relate to their nutritional and ketotic effects. Furthermore, epidemiological studies consistently report increased mortality with the LCD [48]. Whilst the direct effects of the LCD are important, there are also potential indirect safety concerns. As outlined earlier, adoption of the LCD inevitably results in a diminishment of glycogen supplies that may in turn contribute towards fatigue, and reduced athletic performance [30]. Furthermore, given the restricted food choices, potentially reduced pleasure of eating and the fact that the LCD is often adopted in the context of obesity (a highly stigmatized condition in our society), it is important to consider the potential mental and emotional health problems that may occur following adoption of the LCD. Adoption of the LCD may also have a negative impact on relationships, given the central role of food and eating in our social world. As healthcare professionals, we need to be mindful of these issues, and consider the mental health and social scaffold of our patients both prior to suggesting any dietary modification, and at each follow-up opportunity whilst on any diet, including the LCD. There are also ecological and ethical implications of promoting and adopting the LCD for many patients, including the environmental effects of soy and meat production, deforestation and consequent effects on climate change. The socio-economic implications of the LCD include the increased financial expense of the LCD compared with other diets [77], that may preclude the feasibility and affordability of the LCD for lower socio-economic groups within society (who are also most likely to benefit from lifestyle and dietary changes). 

Finally, although this goes well beyond the scope of our concise review, we need to consider the potential for the indirect effects of gut dysbiosis that may stem from adoption of the LCD, with the inherent high intake of fats and protein, typically found in animal-based foods, and relative paucity of carbohydrate- and fibre-laden plant-based foods [78]. Given the emergent data that link our gut microbiota with much of our physiological functioning including the regulation of appetite, metabolism and mental health and wellbeing [79], it is important to consider the impact of any dietary modification on our gut microbiota. This consideration has perhaps, not been given enough attention previously, with too much focus on weight loss per se following implementation of the LCD.

## 7. Conclusions and Future Directions: LCD in the Context of Healthy Lifestyle

Despite decades of research, it is remarkable how little we actually know about the human diet. It is probably fair to say that most of us think we know more than we actually do. There are many reasons why dietary insights remain elusive. These include the inherent difficulties of studying diets in isolation, with every diet containing a complex array of macronutrients and many other variables. There is also the problem of accuracy, with most of us under-estimating our daily caloric intake and problems of self-recall that plague many dietary studies. There are cultural factors, with specific dietary preferences and norms widely variable across global populations. Finally, there are human variables from our own unique genetic and physiological make-up, and the complex array of microbiota within our gut, unique to each individual. Faced with this complex and highly variable set of factors, it is hardly surprising that we have limited knowledge and insights into the optimization of our diets for health. 

Although specific dietary advice exists for certain groups, the vast majority of us are encouraged to follow the standardized government advice regarding a healthy diet, with recommended daily caloric intakes simplified based on sex. However, a more refined approach is required, one that is tailored to the individual, and based on much more sophisticated inputs than sex. As alluded to, the gut microbiota is hugely relevant to our handling of macronutrients, and a future scenario may incorporate individualized gut faecal profiles that inform specific dietary requirements [80]. Genetic profiling may also be included (including gene variants relevant for liver handling of macronutrients for example). A sophisticated dietary algorithm may even incorporate a Bayesian process, in which there is algorithmic learning over time regarding how each individual responds to and assimilates certain diets and macronutrients across a whole range of measures. Such a tool would enable users to make individualized healthy and enjoyable food choices. Algorithmic learning and tailored dietary recommendations would help to ensure the longer-term success and durability of healthy dietary modifications. 

Ultimately, our diet should form an essential component of a healthy lifestyle. From a nutritional perspective, it is important that any dietary intervention contains a proper balance of healthy fats, proteins, carbohydrates, fibre, minerals and vitamins [11]. Due to the problem of longer-term adherence to dietary modifications, any dietary intervention must also be flexible and fit with individual lifestyles, backgrounds, cultures and food preferences [11]. Physical activity and sleep are also important [11]. Based on the available evidence, it is clear that there are specific metabolic benefits (including, for example improved glycaemic control) from the adoption of the LCD. However, these benefits appear short-lived. Conversely, the longer-term benefits of the LCD are disappointing. As a longer-term weight loss strategy, the LCD is simply ineffective in the majority of individuals who adopt it, primarily due to difficulty in sustained adherence to dietary interventions [11]. There are also potential safety concerns with the longer-term application of the LCD, as outlined. Indeed, very few people are able to maintain weight loss following the implementation of any lifestyle intervention, including long-term dietary modifications [11]. 

How can we proceed in the future to optimize our diets for maximal health benefits? There is clearly a need for more large-scale, prospective multi-centre trials on the effects of the LCD. Despite the publication of >100 separate LCD and LFD randomized controlled trials, our understanding of the benefits of the LCD remains limited, at least in part due to heterogeneity amongst the design of different studies. We suggest a bold initiative to explore major dietary approaches such as the LCD on the same scale as research in therapeutics, with a 1–5 year duration and inclusion of >5000 participants. Such a study design would enable the exploration of major primary assumptions (such as remission/prevention of T2D and CVD outcomes), and many secondary assumptions (such as compliance, subgroup analyses [to ascertain the subgroups that are particularly responsive to the LCD and predictors of response] and nutrigenetics). With antecedents of the PREDIMED (Prevenciόn con Dieta Mediterránea) study on the benefits of the Mediterranean diet [81] and the Look AHEAD (Action for Health in Diabetes) study on the benefits of the LFD (as part of an intensive lifestyle intervention) [82], the execution of a large-scale study on the LCD is well overdue. 

As outlined earlier, an individualized and algorithmized approach to diets seems sensible. We also need to change our societal mind-set regarding the purpose of dietary change. Rather than diets perceived simply as a means to lose weight, we need to change our perspective to one in which diets are considered as an essential component of healthy lifestyle, which if followed correctly with other lifestyle measures, will improve our future health outlook, and may even optimize our body weight. Above all, for longer-term adherence, it is essential that whatever the chosen lifestyle strategy (including dietary change), this is practical, feasible and importantly enjoyable for the individual engaged in such behaviour changes. Without enjoyment, any long-term change is destined to fail. Perhaps one reason why dietary and lifestyle changes are so disappointing over the longer term is that the available options are too rigid, and as healthcare professionals, we do not invest enough time in ascertaining practicality, feasibility and enjoyability of suggested behaviour changes for each individual prior to making such recommendations. With such a priori assessments, we can tailor our approach to the individual, and any recommended lifestyle change, dietary or otherwise, will have a greater chance of longer-term success. 

In addition to rigidity, perhaps our traditional approach to diets is also too prescriptive. The available evidence shows that weight loss occurs following a reduction in daily caloric intake, regardless of the macronutrient origin of those calories, although the magnitude of weight loss varies according to the type of macronutrient, and the effects on diet-induced thermogenesis [11]. Therefore, rather than considering the merits of the LCD versus LFD or any other macronutrient change, perhaps our approach should simply be to limit caloric intake, whilst maintaining a healthy and enjoyable diet, and nurturing our gut microbiota with fibre and plant-based foods in the process. The magnitude of limitation in caloric intake (isocaloric balanced diet versus caloric restriction) requires careful tailoring to the individual, with occasional avoidance of caloric restriction, including in the sub-group of patients with T2D and normal-weight and/or older age. (In elderly patients, the adoption of the LCD may worsen the loss of subcutaneous adipose tissue, development of sarcopenia and the risk of renal insufficiency). In the majority of patients with T2D, however, caloric restriction represents an excellent treatment option. In the DIRECT trial (Diabetes Remission Clinical Trial), caloric restriction to between 825 and 853 Kcal/day in adult patients with T2D resulted in nearly 50% remission to a nondiabetic state at 12-months and a mean weight loss of 10 Kg (with a minority achieving >15% body weight loss) [83]. Although the longer-term adherence to caloric restriction of this magnitude may prove challenging to many, and it will be important to review carefully the longer-term follow-up data from the DIRECT cohort, these initial data are indeed promising, and demonstrate proof of concept that caloric restriction is a feasible option, at least in the short-term. 

To conclude, evidence reveals metabolic efficacy of the LCD at least during the short-term. Unfortunately, there are substantial limitations to the LCD that restrict its metabolic efficacy over the longer-term, coupled with potential safety concerns. Compared with the LCD, caloric restriction offers a greater selection of food (including plant- and fibre-based) options with fewer safety concerns (provided maintenance of a balanced diet). Perhaps even relatively modest caloric restriction provides a practical, feasible and enjoyable dietary alternative to the LCD, accomplishable over the longer-term and offering hope for the future. 

## Figures and Tables

**Table 1:** Outline of the potential safety concerns of the LCD.

Safety Concern	Nature of the Problem	Clinical Sequelae
Nutritional Deficiencies	Reduced dietary intake of fibre, minerals, vitamins, trace elements and PUFA [12]; Depleted glycogen stores from restricted carbohydrate intake [43]	Increased mortality from restricted fibre, essential micronutrients and PUFA [48,49,50]; Dyslipidaemia [12]; Bone health and renal calculi [12]; Hypoglycaemia (T1D)
Ketosis	Short-term gastro-intestinal symptoms [12]; Longer-term effects incompletely understood [11] Released Calcium Stores [11]	Vomiting, Diarrhoea and Obstipation; Gastrointestinal reflux [12]; Theoretical Nephrolithiasis and Osteoporosis [11]; Ketoacidosis (rare) [52]
High-Protein Diet	Epidemiological studies show association with dysglycaemia and unfavourable metabolic effects [53,54]; Interventional studies show metabolic benefits [55,56,57,58,59]	Impaired GFR in women with mild renal impairment [11,45,46,47]; Metabolic benefits likely mediated by associated lifestyle changes in interventional studies [60]
Hyperuricaemia	Theoretical risk of excessive conversion of purines from animal proteins [11,61]	Gouty arthritis Uric acid nephrolithiasis
Inflammatory Status	Theoretical promotion of inflammatory effects from a relative increase in dietary saturated fat intake	Evidence is contradictory Data show either no appreciable effect [63] or improvement of inflammatory status [64,65,66,67] in response to the LCD
Mental and Emotional Status	Central role of food and eating within our society	Potential lowering of mood and negative impact on relationships
Ecological and Ethical Concerns	Environmental effects of soy bean and meat production and deforestation	Health implications of climate change
Financial Implications	Increased expense of the LCD [77], and disproportionate financial effects on lower socio-economic groups (reduced affordability and feasibility)	Health implications of financially restricted diet to those groups most likely to benefit from dietary change
Dysbiosis	Relative increase in dietary intake of fats and protein, with a deficiency in dietary fibre intake [78]	Appetitive, immunomodulatory, inflammatory and dysmetabolic sequelae; Impact on mental health and wellbeing [79]

GFR: Glomerular Filtration Rate; LCD: Low-Carbohydrate Diet; PUFA: Polyunsaturated Fatty Acids; T1D: Type 1 Diabetes Mellitus.
